# Eco-epidemiology of *Rickettsia amblyommatis* and *Rickettsia parkeri* in naturally infected ticks (Acari: Ixodida) from South Carolina

**DOI:** 10.1186/s13071-023-06099-z

**Published:** 2024-01-25

**Authors:** Lídia Gual-Gonzalez, Stella C. W. Self, Kia Zellars, Madeleine Meyer, Kyndall C. Dye-Braumuller, Chris L. Evans, Omar Cantillo-Barraza, Myriam W. Torres, Melissa S. Nolan

**Affiliations:** 1 Department of Epidemiology and Biostatistics, University of South Carolina, Columbia, SC USA; 2 Vector-Borne Diseases Laboratory, South Carolina Department of Health and Environmental Control, South Carolina Public Health Laboratory, Columbia, SC USA; 3 Grupo de Biología y Control de Enfermedades Infecciosas, Universidad de Antioquia, Medellín, Colombia

**Keywords:** Rickettsia parkeri, public health, Rickettsia, South Carolina

## Abstract

**Background:**

Spotted fever group *Rickettsia* (SFGR) is the largest group of *Rickettsia* species of clinical and veterinary importance emerging worldwide. Historically, SFGR cases were linked to *Rickettsia rickettsii*, the causal agent of Rocky Mountain spotted fever; however, recently discovered species *Rickettsia parkeri* and *Rickettsia amblyommatis* have been shown to cause a wide range of clinical symptoms. The role of *R. amblyommatis* in SFGR eco-epidemiology and the possible public health implications remain unknown.

**Methods:**

This study evaluated statewide tick surveillance and land-use classification data to define the eco-epidemiological relationships between *R. amblyommatis* and *R. parkeri* among questing and feeding ticks collected across South Carolina between 2021 and 2022. Questing ticks from state parks and feeding ticks from animal shelters were evaluated for *R. parkeri* and *R. amblyommatis* using reverse transcriptase quantitative polymerase chain reaction (RT-qPCR) on pooled samples. A Bayesian multivariable logistic regression model for pool testing data was used to assess associations between *R. parkeri* or *R. amblyommatis* infection and land-use classification variables among questing ticks. The Spearman correlation was used to evaluate the relationship between the two tested pathogens.

**Results:**

The infection prevalence for *R. amblyommatis* was 24.8% (23.4–26.3%) among questing ticks, and 39.5% (37.4–42.0%) among feeding ticks; conversely, for *R. parkeri* it was 19.0% (17.6–20.5%) among questing ticks and 22.4% (20.3–24.5%) among feeding ticks. A negative, refractory correlation was found between the species, with ticks significantly more likely to contain one or the other pathogen, but not both simultaneously. The Bayesian analysis revealed that *R. amblyommatis* infection was positively associated with deciduous, evergreen, and mixed forests, and negatively associated with hay and pasture fields, and emergent herbaceous wetlands. *Rickettsia parkeri* infection was positively associated with deciduous, mixed, and evergreen forests, herbaceous vegetation, cultivated cropland, woody wetlands, and emergent herbaceous wetlands, and negatively associated with hay and pasture fields.

**Conclusions:**

This is the first study to evaluate the eco-epidemiological factors driving tick pathogenicity in South Carolina. The negative interactions between SFGR species suggest the possible inhibition between the two pathogens tested, which could have important public health implications. Moreover, land-use classification factors revealed environments associated with tick pathogenicity, highlighting the need for tick vector control in these areas.

**Graphical Abstract:**

**Figure:**
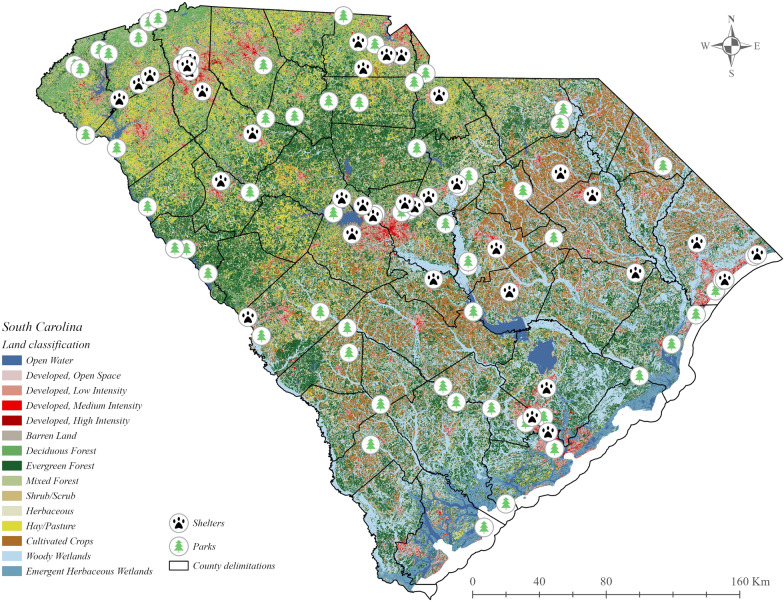

**Supplementary Information:**

The online version contains supplementary material available at 10.1186/s13071-023-06099-z.

## Background

*Rickettsia* spp. within the spotted fever group (SFGR) are a large group of tick-borne intracellular bacteria comprising several clinical and veterinary important species, and pose a major re-emerging public health concern worldwide [1]. SFGR pathogenicity varies from severe to mild disease: *Rickettsia rickettsii*, the causal agent of Rocky Mountain spotted fever (RMSF), is considered the most pathogenic species, while other species such as *Rickettsia parkeri* result in a similar, often less severe disease [2]. The main *R. rickettsii* vectors in the United States are *Dermacentor variabilis*, *Dermacentor andersoni*, and *Rhipicephalus sanguineus* [3, 4]. *Rickettsia parkeri* is also an important disease-causing agent, first described in 2004 among *Amblyomma maculatum* ticks [2]. The clinical symptoms associated with this species are considered different from those caused by *R. rickettsii*; however, unless molecular diagnostic testing is performed, the serological tests cannot be used to confirm species, and therefore clinical confirmation is based entirely on symptomology. One species in particular, *Rickettsia amblyommatis* (previously *Candidatus* R. amblyommii), has an undefined pathogenicity that has been highly debated by the clinical community. Some propose this species to be pathogenic, while others argue that it is non-pathogenic [5, 6]. Clinical diagnostic tests traditionally do not distinguish between SFGR species, and thus the ability to study veritable *R. parkeri* or other *Rickettsia* spp. clinical cases is challenging, contributing to the ongoing lack of knowledge regarding the pathogenic potential of other species.

Laboratory mice and guinea pig studies have demonstrated that *R. amblyommatis* can cause clinical symptoms under experimental conditions [7, 8], suggesting that mild human cases could be related to this species. Serological evidence in patients with a history of disease suggests that humans can become infected and produce high antibody titers against *R. amblyommatis* [9]. In South Carolina, USA, a pediatric hospitalization case was reported in the late 2010s (Chris Evans, personal communication, May 4, 2022) further suggesting the pathogenic potential of this species. We hypothesize that a portion of SFGR clinical cases linked to *R. amblyommatis* are misdiagnosed due to *Rickettsia* spp. antibody cross-reactivity, given the large number of RMSF cases in areas with high *R. amblyommatis*-infected tick prevalence [10]. For instance, *R. rickettsii* has been found in approximately 0.1% of ticks, while *R. amblyommatis* can be found present in more than 50% of ticks, indicating a greater *R. amblyommatis* exposure risk [11, 12]. Given the potential for *R. amblyommatis* to cause human disease, the need for targeted tick pathogen surveillance is warranted.

*Rickettsia amblyommatis* was first isolated from an *Amblyomma americanum* tick collected in Tennessee in 1974, and repeatedly detected in naturally infected *A. americanum* ticks throughout the USA [13, 14]. North Carolina is one of the states with the highest RMSF incidence [15]; however, since *R. rickettsii* is rarely found in ticks, investigators have postulated that some cases could be due to *R. amblyommatis* infection [9, 16, 17]. A tick surveillance initiative in North Carolina detected *R. amblyommatis* in 37% of *A. americanum* tick pools and 8.7% of *D. variabilis* tick pools but found *R. rickettsii* in 0% of pools, indicating that those bitten by ticks in North Carolina are far more likely to encounter *R. amblyommatis* when bitten [9]. Similarly, in another study, almost 55% of *A. americanum* ticks were *R. amblyommatis*-positive across all tested sites in Chatham County, NC [16]. An Oklahoma study detected *R. amblyommatis* in dogs exposed to environmental ticks (> 90% *A. americanum*, 3% *A. maculatum*, and 6% *D. variabilis*) [18]. Therefore, *R. amblyommatis* has been the most widely detected SFGR species across several studies investigating ticks attached to humans, although limited clinical signs have ever been described related to this species [17, 19]. In one case, a *R. amblyommatis*-positive tick was removed from the skin of a patient who developed a macular rash, although no other clinical signs were detected once the patient began doxycycline treatment [20]. Nevertheless, eco-epidemiological studies suggest that *R. amblyommatis* could be related to milder cases, since most SFGR cases happen in *A. americanum* high-prevalence areas (this tick is not considered a primary vector for *R. rickettsii*), and most patients with SFGR antibodies show higher titers against *R. amblyommatis* than any other *Rickettsia* spp. [11].

Despite the circumstantial evidence, little is known about the role of *R. amblyommatis* in SFGR cases and the eco-epidemiological factors associated with exposure. Under laboratory conditions, there have been several findings evaluating *Rickettsia* species interactions. One study found that *R. amblyommatis*-positive ticks co-feeding with *R. parkeri*-infected ticks are less likely to acquire the former, suggesting a possible inhibitory effect [21]. Other investigations have found that previous exposure to other *Rickettsia* spp. could reduce the severity of disease caused by *R. rickettsii*; however, no clear epidemiological evidence has been described in humans yet [22]. Thus, there is a need for epidemiological studies to unravel these pathogen interactions.

The South Carolina landscape promotes outdoor recreational activities at multiple state parks, creating tick exposure opportunities and subsequently SFGR exposure opportunities [23]. In 2020, a tick surveillance initiative was implemented in South Carolina where questing ticks were collected from state parks and public submissions, and host-attached ticks were collected from South Carolina animal shelters [24]. Given that South Carolina reports a moderate number of SFGR cases, and *A. americanum* is the most commonly found questing tick, *R. amblyommatis* is likely to be present in ticks collected throughout the state. These ticks should be evaluated to better understand the epidemiological characteristics of this infection [15, 24]. To evaluate the eco-epidemiological risk factors associated with *R. amblyommatis* in metastriate ticks, this study aims to investigate *R. amblyommatis* and *R. parkeri* distribution and prevalence utilizing reverse transcriptase quantitative polymerase chain reaction (RT-qPCR) and land-use classification environmental factors using a Bayesian analysis across the state parks of South Carolina.

## Methods

### Study area and sampling locations

South Carolina is a coastal state in the Southeastern USA, with a total area of 82,933 km^2^, and is bordered by North Carolina to the north and northeast, Tennessee to the northwest, Georgia to the southwest, and the Atlantic Ocean to the southeast. Slightly over 5 million residents live in the state, with 45% living within the counties encompassing the major metroplexes: Charleston, Columbia, Greenville, and Myrtle Beach [25]. The state can be divided into three main areas, the Blue Ridge Mountain in the northwestern corner (rising to 1085 m elevation), the Piedmont from the mountains to the Sandhills in the Southeastern Plains (90 to 365 m elevation), and the Coastal Plain comprising Southern and Middle Atlantic regions (from sea level to 90 m elevation) delimited by the Sandhills and Coastal zone. The state has a subtropical climate, with hot, humid summers (average 23–33 °C, 15 days precipitation in July) and mild winters (average 3–15 °C, 9 days precipitation in January) [26]. Vegetation varies from woodlands in the Blue Ridge region, row crops in the Piedmont region with some loblolly pine forest, and gum, live oaks, cypresses, and magnolias across the coastal plain. Most wildlife is spread across the state, but some species follow geographical boundaries between the Coastal Plain and Piedmont [23]. Wildlife is best represented by white-tailed deer in the Piedmont and Coastal Plain, woodchucks and red squirrels found in the Blue Ridge, and American beaver, wild turkey, red foxes, and European wild pigs across the state.

For this study, we used metastriate ticks (Acari: Ixodidae) collected from January 2021 to December 2022 from a statewide tick surveillance effort recently implemented in South Carolina [24]. The ticks originated from either animal shelter submissions (host-attached ticks removed at the time of admission to one of 20 participating humane shelters statewide) or state park-based surveillance (collection of questing ticks using 10 CO_2_-baited traps, body-found ticks from the staff performing collections, and 50 m^2^ density dragging at each of 39 state parks) [27]. This program aims to elucidate tick-borne pathogen distribution across the state of South Carolina by collecting state park questing ticks, and tallying animal shelter feeding ticks.

### Pathogen testing

Ticks were morphologically identified by species, sex, and life stage using dichotomous taxonomical keys by Keirans and Litwak [28], Egizi [29], and Clifford and Anastos [30]. Only metastriate ticks were selected and included, and non-metastriate ticks were kept in a 70% ethanol solution for future usage. After identification, host-attached ticks and questing ticks were bisected longitudinally and used for DNA extraction. Questing ticks were pooled by species, sex, life stage, collection method, collection date, and location in the following pool sizes: three for adults, five for nymphs, and a single pool for all the larvae. Host-attached ticks were tested individually. Each testing pool or individual tick was homogenized using 180 µl Qiagen Buffer ATL and two 5-mm stainless-steel beads using a bead homogenizer (TissueLyser, Qiagen, Germantown, MD, USA). DNA was extracted from the homogenized pools using QIAmp 96 DNA QIAcube Mini Kit (Qiagen, Germantown, MD, USA) on the QIAcube HT workstation (Qiagen, Germantown, MD, USA), following the manufacturer’s instructions. Gene amplification was performed to evaluate *R. amblyommatis* and *R. parkeri* DNA presence. DNA detection was performed by RT-qPCR using validated primers and probes for the outer membrane B gene *ompB* specific to *R. amblyommatis* and *R. parkeri* (Additional file 1: Table S1). The amplifications were performed using the QuantStudio 5 Real-Time PCR system (Applied Biosystems, Foster City, CA, USA) following the manufacturer’s instructions. Cycling conditions were 95 °C for 3 min and a two-step cycling for 40 cycles. Samples were considered positive for *R. amblyommatis* or *R. parkeri* when the cycle threshold (Ct) value was ≤ 40.

### Statistical analysis

Descriptive statistics were performed to determine the pathogen distribution across tick species between questing and host-attached ticks, life stage, feeding status, and sampling method. *Rickettsia amblyommatis* and *R. parkeri* prevalence and 95% confidence intervals (CIs) were calculated for questing and attached ticks from PCR-positive pools using the Epitools^®^ prevalence calculator for pooled samples (Ausvet, Fremantle, WA, Australia). This method accounts for different pool sizes to estimate prevalence [31]. A Spearman correlation was performed to evaluate the co-presence of *R. amblyommatis* and *R. parkeri* within pools. Additionally, the presence of *R. amblyommatis* and *R. parkeri* was mapped to evaluate the overlapping geographical distribution between the two pathogen species. For visualization, a layer including the ecological regions from South Carolina was obtained from the US Environmental Protection Agency [32].

Bayesian logistic regression models for group testing data [33] were used to evaluate the association between *R. amblyommatis* and *R. parkeri* presence and tick species, collection method, life stage, and land-use classification parameters. The Bayesian regression models were fitted for questing ticks collected in South Carolina state parks only due to the unknown geographical origin of the animals hosting attached ticks. The land-use classification variables were obtained from raster data from the National Land Cover Database from 2019 [34]. Values for percent land-use classification variables at each park were obtained to create a 10-km radius buffer within the park extension and summarized as a categorical raster to provide numerical values of the percentage area covered. The description of each land-use classification variable can be found in Additional file 1: Table S2. The values were exported to a data file and incorporated into the final dataset. All variables were evaluated using an unadjusted model and were considered to be included in the final adjusted model. Variables included in the final model were chosen using step-wise variable selection and model convergence. The models were run for 10,000 Markov chain Monte Carlo (MCMC) iterations with the first 5999 iterations discarded as burn-in. Convergence was assessed with trace plots (Additional file 2). A variable was considered to have a statistically important relationship with infection status if the 95% equal-tail credible interval for the variable’s regression coefficient did not contain 0. Statistical analysis and data visualization were performed using Epitools^®^ (Ausvet, Fremantle, WA, Australia), RStudio, R version 4.1.3 (Free Software Foundation, Boston, MA, USA), and ArcGIS Pro 2.8.3 (Esri, Redlands, CA, USA).

## Results

Between January 2021 and December 2022, a total of 4412 metastriate ticks were collected or submitted from South Carolina state parks (*n* = 2909) and animal shelters or humane societies (*n* = 1503), respectively. Of the total ticks collected, 2556 were adults, 1611 were nymphs, and 245 were larvae. There were five metastriate species collected: 3364 (76.2%) *A. americanum*, 799 (18.1%) *D. variabilis*, 195 (4.4%), *A. maculatum*, 44 (1.0%) *R. sanguineus*, and 10 (0.2%) *Haemaphysalis longicornis*. As shown in Table 1, only two species were obtained from parks—2188 (49.36%) were collected using CO_2_ traps, followed by 619 (14.0%) collected from density dragging. Animal shelters submitted ticks representing all five species. Pathogen point prevalence estimates among questing ticks—irrespective of species—were 24.8% (95% CI 23.4–26.3%) for *R. amblyommatis* and 19.0% (95% CI 17.6–20.5%) for *R. parkeri*. Among all feeding ticks, the estimated point prevalence was 39.5% (95% CI 37.4–42.0%) for *R. amblyommatis* and 22.4% (95% CI 20.3–24.5%) for *R. parkeri*.

**Table 1:** Classification of collected ticks by species, life stage, sampling method, and feeding status

Questing ticks								
Species	Life stage					Sampling method		
	Larva	Nymph	Adult females	Adult males	Total	Dragging	CO_2_ traps	Found on body/bite
*Amblyomma americanum*	243	1581	604	467	2895	606	2187	96
*Dermacentor variabilis*	0	0	8	6	14	13	1	0
Total	243	1581	612	473	2909	619	2188	96

Host-attached ticks								
Species	Life stage					Feeding status		
	Larva	Nymph	Adult females	Adult males	Total	Fully fed	Partially fed	Unfed
*Amblyomma americanum*	1	1	261	206	469	18	93	358
*Amblyomma maculatum*	0	0	90	105	195	4	24	167
*Dermacentor variabilis*	1	10	394	380	785	49	168	568
*Haemaphysalis longicornis*	0	4	6	0	10	0	7	3
*Rhipicephalus sanguineus*	0	15	20	9	44	0	30	14
Total	2	30	771	700	1503	71	322	1110

A significant moderate negative correlation between *R. amblyommatis* and *R. parkeri* positivity was found for shelter submissions (*ρ* = −0.142), for park collections (*ρ* = −0.422), for *A. americanum* (*ρ* = −0.301), and for *R. sanguineus* (*ρ* = −0.38) pools. Among life stages, nymphs showed the strongest negative correlation between *R. amblyommatis* and *R. parkeri* positivity (*ρ* = −0.388). Questing ticks showed the strongest negative correlation (*ρ* = −0.422) (Table 2).

**Table 2:** *Rickettsia amblyommatis* and *R. parkeri* positivity correlation by tick species, life stage, and location

	*N* pools^a^ (%)	*R. amblyommatis*-positive pools *N* (%)	*R. parkeri*-positive pools *N* (%)	Spearman’s rank correlation (*ρ*)	*P*-value
*Amblyomma americanum*	879	543 (61.8)	240 (44.2)	−0.301	< 0.0001
*Amblyomma maculatum*	194	11 (5.7)	120 (61.8)	−0.037	0.61
*Dermacentor variabilis*	784	227 (28.9)	149 (19.0)	−0.015	0.67
*Haemaphysalis longicornis*	10	1 (10.0)	2 (20.0)	−	−
*Rhipicephalus sanguineus*	44	21 (47.7)	6 (13.6)	−0.380	0.01
Larvae	20	8 (40.0)	10 (50.0)	−0.408	0.07
Nymph	254	122 (48.0)	109 (42.9)	−0.388	< 0.0001
Female	870	390 (44.8)	205 (23.6)	−0.097	0.004
Male	767	283 (36.9)	193 (25.2)	−0.232	< 0.0001
Adults	1637	673 (41.1)	398 (24.3)	−0.189	< 0.0001
Parks (questing ticks)	423 (22.1)	214 (50.6)	183 (43.3)	−0.422	< 0.0001
Shelters (host-attached ticks)	1490 (77.9)	589 (39.5)	334 (22.4)	−0.142	< 0.0001

^a^Pools that could not be tested for both pathogens are not included

**Fig. 1:**
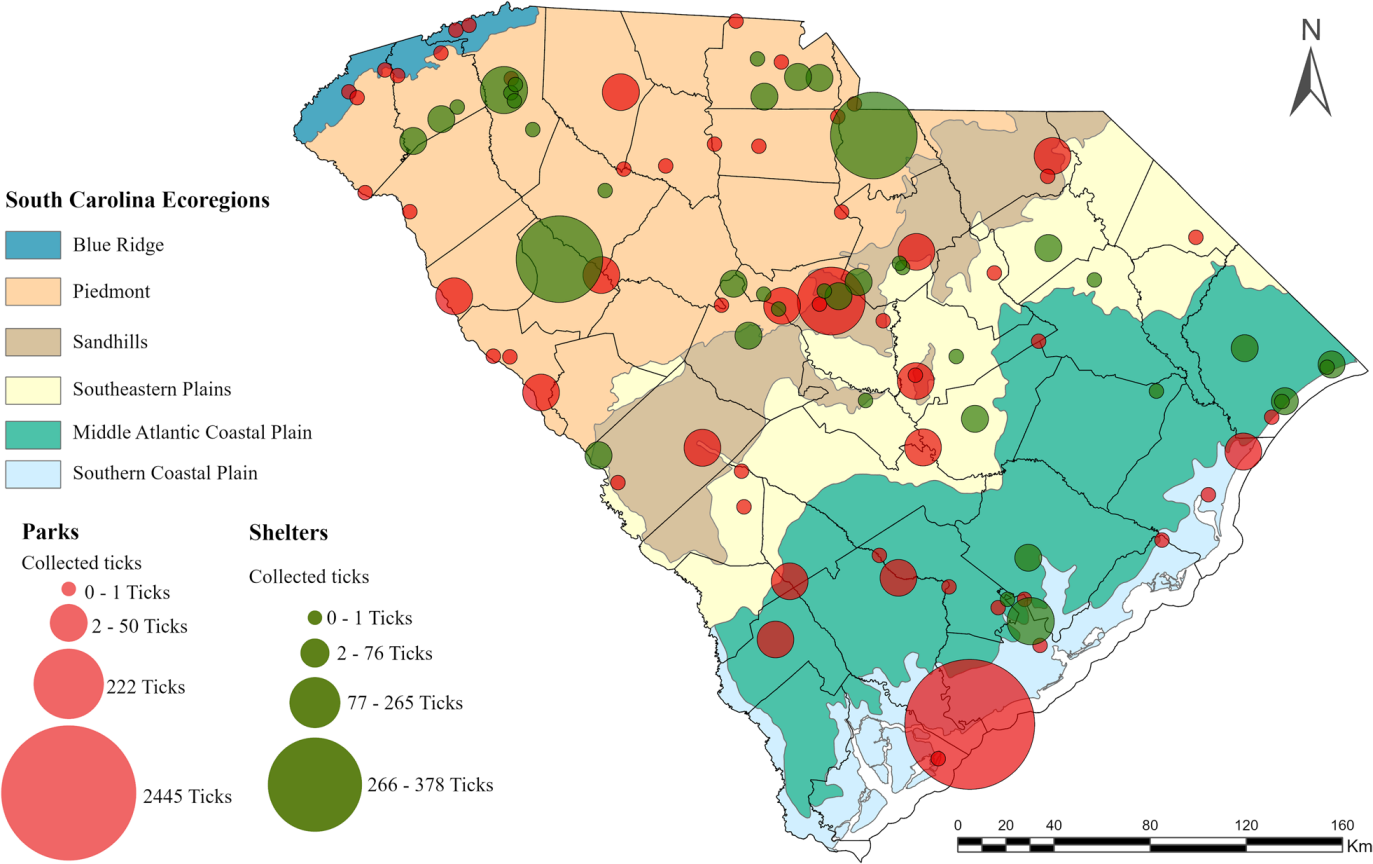
Distribution and number of ticks collected statewide between January 2021 and December 2022

**Fig. 2:**
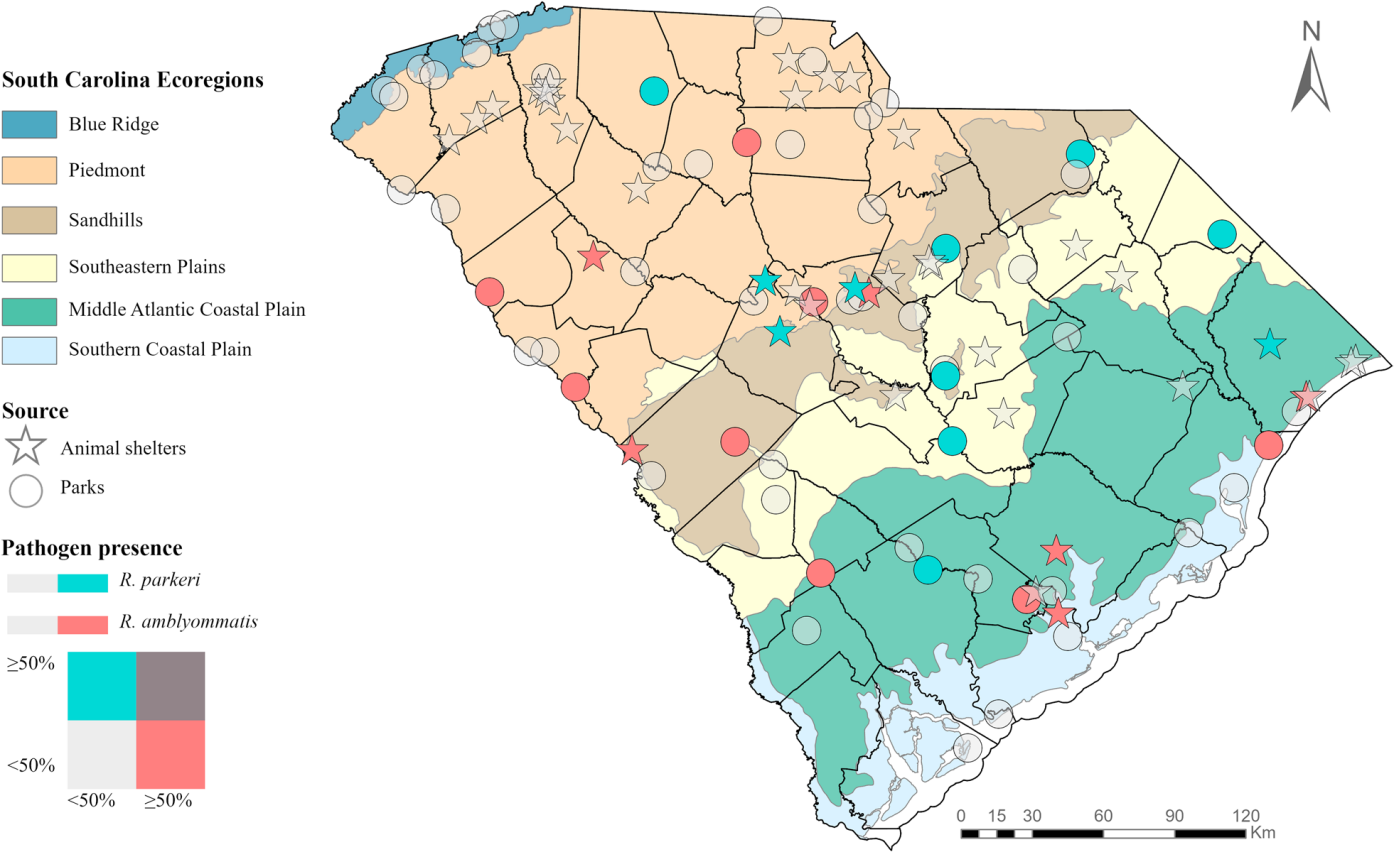
Bivariate distribution of *R. parkeri* and *R. amblyommatis* metastriate tick positivity by host-seeking status. *Note: Questing ticks were collected at state parks, and host-attached ticks were collected from animal shelters and humane societies

Ticks were collected from every region of the state, although the Blue Ridge region yielded the fewest ticks. Two state parks, Edisto State Park located in the Southern Coastal Plains and Sesquicentennial State Park located in the Sandhills, produced the largest volume of metastriate ticks. Similarly, two animal shelter locations, both located in the Piedmont region, produced the highest volume of host-attached ticks (Fig. 1). Some geographical overlapping existed between *R. parkeri*- and *R. amblyommatis*-infected ticks (Fig. 2); however, one or the other was typically predominant, and these were not present simultaneously in 50% or more of the pools from the same region. *Rickettsia amblyommatis* was generally collected throughout the southern part of the state, whereas *R. parkeri*-positive ticks were collected in the more northern part of the state.

The Bayesian logistic regressions yielded statistically important variables that showed negative and positive associations with *R. amblyommatis* and *R. parkeri* infection (Table 3). In both models, female ticks were more likely to be positive for *R. amblyommatis* than males or immature ticks (odds ratio [OR]: 1.5, 95% CI 1.10–2.03; adjusted OR [aOR]: 1.43, 95% CI 1.03–1.99). Ticks collected through dragging were more likely to be positive for *R. amblyommatis* than ticks collected through CO_2_ traps or found on the body (OR: 1.53, 95% CI 1.11–2.06; aOR 1.55, 95% CI 1.11–2.15). In the unadjusted model, *R. amblyommatis* was more likely to be infecting ticks in the presence of deciduous forest (OR: 1.05, 95% CI 1.02–1.08), evergreen forest (OR: 1.06, 95%CI:1.03–1.08), and mixed forest (OR: 1.08, 95% CI 1.05–1.13). Both the unadjusted and adjusted models showed that *R. amblyommatis* infection was less likely to occur in the presence of emergent herbaceous wetlands (OR: 0.98, 95% CI 0.97–0.98; aOR: 0.90, 95% CI 0.83–0.98). In contrast, both unadjusted and adjusted models indicated that *R. parkeri* infection was positively associated with herbaceous vegetation (OR: 1.07, 95% CI 1.01–1.13; aOR: 1.61, 95% CI 1.22–2.15), cultivated crops (OR: 1.23, 95% CI 1.12–1.37; aOR: 19.93, 95% CI 4.28–69.12), and woody wetlands (OR: 1.03, 95% CI 1.01–1.05; aOR: 1.89, 95% CI 1.17–2.66). In the unadjusted model, *R. parkeri* was negatively associated with hay and pasture fields (OR: 0.86, 95% CI 0.78–0.95). In the adjusted model, only *R. parkeri* was positively associated with the presence of deciduous forest (aOR: 2.18, 95% CI1.51–2.95), mixed forest (aOR: 2.94, 95% CI 1.65–4.96), and emergent herbaceous wetlands (aOR: 2.01, 95% CI 1.51–2.61).

**Table 3:** Adjusted and unadjusted Bayesian logistic regression for pooled data evaluating *R. amblyommatis* and *R. parkeri* positivity prediction through environmental factors

Description	*R. amblyommatis* positivity						*R. parkeri* positivity					
	OR	95% ETCI^a^		aOR^b^	95% ETCI^a^		OR	95% ETCI^a^		aOR^c^	95% ETCI^a^	
*D. variabilis*	2.30	0.61	7.86	0.53	0.08	2.67						
Female	1.50	1.10	2.03	1.43	1.03	1.99	1.14	0.74	1.69	0.73	0.73	1.71
Drag	1.53	1.11	2.06	1.55	1.11	2.15	1.17	0.78	1.69	1.11	0.66	1.67
Deciduous forest	1.05	1.02	1.08	0.93	0.85	1.00	1.00	0.96	1.04	2.18	1.51	2.95
Evergreen forest	1.06	1.03	1.08	0.96	0.86	1.07	0.95	0.91	0.98	2.11	1.20	3.23
Mixed forest	1.08	1.05	1.13	0.94	0.85	1.06	1.02	0.97	1.06	2.94	1.65	4.96
Herbaceous vegetation	1.09	1.04	1.14	0.72	0.56	0.92	1.07	1.01	1.13	1.61	1.22	2.15
Hay/pasture fields	0.77	0.71	0.83	0.96	0.49	1.83	0.86	0.78	0.95	0.85	0.07	2.29
Cultivated cropland	0.97	0.84	1.09	0.97	0.80	1.16	1.23	1.12	1.37	19.93	4.28	69.12
Woody wetlands	1.01	0.99	1.02	0.93	0.86	1.00	1.03	1.01	1.05	1.89	1.17	2.66
Emergent herbaceous wetlands	0.98	0.97	0.98	0.90	0.83	0.98	0.99	0.98	1.00	2.01	1.51	2.61

^a^Equal-tailed credible interval

^b^Model adjusted for *D. variabilis*, female, drag, deciduous forest, evergreen forest, mixed forest, herbaceous vegetation, hay/pasture fields, cultivated cropland, woody wetlands, and emergent herbaceous wetlands

^c^Model adjusted for *D. variabilis,* female, drag, deciduous forest, evergreen forest, mixed forest, herbaceous vegetation, hay/pasture fields, cultivated cropland, woody wetlands, and emergent herbaceous wetlands

## Discussion

This is the first study evaluating the ecological factors driving *R. amblyommatis* and *R. parkeri* infection among metastriate ticks in South Carolina and one of a few in the Southeastern USA. In this analysis, we estimated an overall *R. amblyommatis* prevalence of 24.8% among questing ticks and prevalence of 39.5% among host-attached ticks. In comparison, *R. parkeri* infection was slightly lower, with 19.0% prevalence among questing ticks and 22.4% among host-attached ticks. This analysis found a negative correlation between *R. amblyommatis* and *R. parkeri* presence within tested tick pools, potentially suggesting an antagonistic relationship between these two species. Ticks were less likely to be *R. amblyommatis*-positive in the presence of emergent herbaceous wetlands, whereas *R. parkeri*-positive ticks were more likely in herbaceous vegetation, cultivated crops, and woody wetlands. In the unadjusted models, both infections were less likely to be present in hay or pasture fields.

Environmental characteristics mediate the sustained transmission of tick-borne pathogens, and knowing the underlying variables associated with vector–pathogen presence can aid in vector control decision-making. It has been previously identified that increased suitable habitat presence correlates with greater species diversity, and thus enzootic cycles have more opportunity to propagate [35]. Considering that deciduous and mixed forests (environments home to greater species diversity) were both positively associated with *R. amblyommatis* and *R. parkeri* presence, sylvatic cycles, not evaluated in this analysis, likely serve as pathogen influencers in these tick populations [36]. In particular, hay pasture fields (environments with lower species diversity and potential acaricide use) [37] showed a negative association with *R. amblyommatis* and *R. parkeri* tick infection. On the contrary, cultivated cropland was positively associated with *R. parkeri* tick infection. Similarly, the scientific literature suggests that rural areas are associated with greater SFGR human risk [38, 39]; rural habitats typically sustain sylvatic transmission foci. Lastly, *R. amblyommatis* infections were less likely in emergent herbaceous wetlands. These landscapes are inhabited mostly by reptile, amphibian, and bird species, which are poorer propagators of tick-borne bacteria, potentially explaining the negative statistical association with *R. amblyommatis* in this study [40–43]. Future studies should aim for a One Health approach to evaluate interactions between hosts, environment, and ticks to better understand the pathogen distributions.

*Rickettsia amblyommatis* has been previously described in 17 countries and within 27 states in the USA [10]. First identified in *A. americanum*, the geographical distribution of this tick species in the USA overlaps with *R. amblyommatis*, further supported by high infection rates among this tick species [10, 44]. *Amblyomma americanum* is a known human-biting species, implicated in the transmission of several tick-borne pathogens [45]. From the tested pools in this study, *R. amblyommatis* was found in 61.8% of *A. americanum*, which is comparable to previously reported infection rates in questing ticks in SC and neighboring states ranging from 29 to 87% [46–49]. Given its wide distribution and high tick infection rates, *R. amblyommatis* could play an important role in SFGR epidemiology. In humans, seroconversion specific to *R. amblyommatis* has been observed, with some individuals expressing greater antibody titers to this than to other SFGR species, suggesting that humans might be exposed to *R. amblyommatis* at greater rates [50, 51]. Human pathogenicity from this species has been hypothesized and demonstrated in animal models, and anecdotal evidence suggests that *R. amblyommatis* infections could be implicated in undetermined febrile illness [9, 20]. Given the difficulty in determining species using an indirect fluorescent antibody (IFA) test due to cross-reactivity between *Rickettsia* spp., some authors suggest that probable mild RMSF cases could be caused by *R. amblyommatis*, and thus future studies should evaluate the pathogenicity of this species [9, 50, 51].

Questing ticks were collected from across the state; however, the majority of ticks were collected in the Southern Coastal Plains region compared to the Blue Ridge and the Piedmont regions. This could be explained by biodiversity differences across regions affecting tick populations. Moreover, CO_2_ traps, placed deeper in the forest compared with density dragging performed on the trails, are better suited for *A. americanum*, the predominant species collected [52]. Nonetheless, additional drivers such as anthropogenic activities and species diversity likely affect tick density in the Southern Coastal Plain region [53]. Conversely, greater numbers of host-attached ticks were collected in the Piedmont region. Additionally, more tick species were submitted that were host-attached than were collected in state parks, likely due to the low efficiency in capturing a diversity of species through CO_2_ traps and dragging. Interestingly, the collection methods were associated with *R. amblyommatis* positivity but not *R. parkeri* positivity. Ticks collected through dragging were more likely to be *R. amblyommatis*-positive ticks than those collected by CO_2_ traps; this suggests that *R. amblyommatis* infection impacts questing behavior, potentially affecting human SFGR exposure [54]. Despite this finding, the relationship between *R. amblyommatis* vector infection, questing behavior, and its epidemiological implications remains unknown.

A negative correlation was observed between *R. amblyommatis* and *R. parkeri* infections among *A. americanum* and *R. sanguineus* ticks. This negative correlation was seen for all life stages except larvae, suggesting the existence of mechanisms preventing co-infection between *Rickettsia* species. We hypothesize that *R. amblyommatis* infection acts as an inhibitory system for other *Rickettsia* spp. transmission, previously observed in laboratory conditions [21, 55, 56]. A study revealed that both *Rickettsia rhipicephali* and *Rickettsia montanensis* have the ability to inhibit *R. parkeri* during transovarial transmission in *D. variabilis*. It was reported that ticks infected with either species would not permit the transovarial transmission of the second species, indicating that ticks will not maintain both species equally, impacting the SFGR eco-epidemiology [55]. Although our results were not significant for negative correlation among larvae, a similar trend was observed (alpha = 0.1), which supports these findings. The current analysis supports the premise of naturally occurring inhibition of *R. parkeri* infection among *R. amblyommatis*-positive ticks during co-feeding. A laboratory evaluation confirmed that among *A. americanum*, *R. parkeri* infection was less likely to occur during co-feeding in *R. amblyommatis*-infected ticks than pathogen-free ticks [21]. In the analysis, this was not observed among *A. maculatum*, the most important *R. parkeri* vector. Unlike in *A. americanum,* the inhibitory phenomenon has not been observed in *A. maculatum*, and co-infections have been described, which could explain why *A. maculatum* is considered the predominant *R. parkeri* vector [56]. Interestingly, *A. maculatum* had the lowest *R. amblyommatis* positivity among host-attached ticks, and because co-feeding was not evaluated, the host-tick-pathogen interaction needs to be further evaluated. Despite *R. amblyommatis* being considered mildly pathogenic, these findings could reveal the mechanisms driving the inhibition of SFGR transmission and the public health implications of this species.

Some study limitations are worth mentioning. First, animal shelter-submitted ticks could not be evaluated for co-feeding as there was no information on which animals the ticks were coming from, nor which ticks were sharing a host. Therefore, the prevalence estimates, or the associations between pathogen presence and host-attached tick species could be overestimated. Second, due to the unknown location where the animal host became infested with ticks, the environmental land-use classification variables analysis was only performed for questing ticks, reducing the number of ticks included in the final analyses. Third, not all the collected ticks could be analyzed for *R. amblyommatis* or *R. parkeri* due to the low quantity of DNA extracted in some tick pools, therefore the correlation analysis was limited to high-quality DNA present specimens which are not representative of all populations. Finally, there was a disproportionate distribution of tick species despite systematic sampling methods used, with over 75% being *A. americanum*, which reduces the representation of some findings to other tick species. Despite these limitations, this is the first eco-epidemiological evaluation of *R. amblyommatis* and *R. parkeri* in South Carolina, and therefore these results remain meaningful ecological and epidemiological findings. Future directions should aim at including animal host densities, blood meal analysis, and possible tick species interactions within hosts, to better understand how the environmental impact on animal host and tick vector species can influence pathogen distribution.

## Conclusions

In conclusion, *R. amblyommatis* and *R. parkeri* are widely distributed tick-borne pathogens across South Carolina that pose human health concerns. Ecological drivers, particularly those in deciduous and mixed forests and agriculture/livestock land, were found to play crucial roles in *Rickettsia* spp. distribution statewide. Despite limited clinical evidence of *R. amblyommatis* pathogenicity, this SFGR species has the potential to play a regulatory role through a possible inhibitory interaction with *R. parkeri*. Ticks carrying *R. amblyommatis* are suggested to be less likely to be infected with and possibly transmit fewer SFGR pathogens; thus this species has major public health interest. Given the higher rates of *A. americanum* across the state and the low presence of *R. parkeri* cases described, we hypothesized that higher prevalence of *R. amblyommatis*-infected *A. americanum* could translate to lower SFGR transmission among humans. Finally, the differences in environmental factors and *Rickettsia* species positivity suggest that enzootic cycles are influenced by microclimate conditions. These suggestions should be further explored using a One Health approach to better understand the possible relationships between SFGR species, tick populations, animal hosts, and the environmental factors affecting their distribution.

### Supplementary Information

**Additional file 1: Table S1.** Primers and probes used for *R. amblyommatis* and *R. parkeri* amplification. **Table S2.** Land-use classification name descriptions.

**Additional file 2:** Markov chain Monte Carlo analysis technical results.

## Data Availability

The data supporting the findings of the study must be available within the article and/or its supplementary materials, or deposited in a publicly available database.

## Abbreviations

aOR: Adjusted odds ratio
CI: Confidence interval
CO2: Carbon dioxide
ETCI: Equal tail credible interval
IFA: Indirect fluorescent antibody assay
MCMC: Markov chain Monte Carlo
NC: North Carolina
OR: Odds ratio
PCR: Polymerase chain reaction
qPCR: Quantitative polymerase chain reaction
RMSF: Rocky Mountain spotted fever
RT-PCR: Real time polymerase chain reaction
SC: South Carolina
SFGR: Spotted fever group *Rickettsia sp.*
