# Supplementary material for: Eco-epidemiology of Rickettsia amblyommatis and Rickettsia parkeri in naturally infected ticks (Acari: Ixodida) from South Carolina
Source: Parasit Vectors. 2024 Jan 25;17:33. doi: 10.1186/s13071-023-06099-z (PMC10811935; doi:10.1186/s13071-023-06099-z)
Supplement: Supplementary file 1 — Additional file 1: Table S1. Primers and probes used for R. amblyommatis and R. parkeri amplification. Table S2. Land-use classification name descriptions. [file 13071_2023_6099_MOESM1_ESM.docx]

Table S1. Primers and probes used for *R. amblyommatis* and *R. parkeri* amplification.

| Name | Target gene | | Sequence (5’ – 3’) | Size (bp) | Reference |
| --- | --- | --- | --- | --- | --- |
| *R. amblyommatis* | | | | | |
| Ra477F | *ompB* | GGTGCTGCGGCTTCTACATTAG | | 141 | ^53^ |
| Ra618R |  | CTGAAACTTGAATAAATCCATTAGTAACAT | |  |  |
| Ra532P |  | HEX-TCCTCTTACACTTGGACAGAATGCT-BHQ2 | |  |  |
| *R. parkeri* | | | | | |
| Rpa129F | *ompB* | CAAATGTTGCAGTTCCTCTAAA | | 96 | ^54^ |
| Rpa224R |  | AAAACAAACCGTTAAAACTACCG | |  |  |
| Rpa188P |  | 6-FAM-CGCGAAATTAATACCCTTATGAGCAGCAGTCGCG-BHQ-1 | | 24 |  |

Table S2. Land-use classification name descriptions.

| Class | Description |
| --- | --- |
| Open water | Water. Less than 25% vegetation or soil cover. |
| Developed, Open Space | Less than 20% constructed and impervious surfaces |
| Developed, Low Intensity | Between 20 and 49% constructed and impervious surfaces. |
| Developed, Medium Intensity | Between 50 and 79% constructed and impervious surfaces |
| Developed, High Intensity | Between 80% and 100% constructed and impervious surfaces. |
| Barren Land | Rock, Sand, or Clay. Less than 15% vegetation. |
| Deciduous Forest | Trees over 5 meters tall make up over 20% of the vegetation. Seasonal changes in foliage in more than 75% of trees. |
| Evergreen Forest | Trees over 5 meters tall make up over 20% of the vegetation. Seasonal maintenance in foliage in more than 75% of trees. |
| Mixed Forest | Trees over 5 meters tall make up over 20% of the vegetation. Deciduous or evergreen trees are less than 75% of trees. |
| Shrub/Scrub | Shrubs. Trees less than 5 meters tall, shrub canopy greater than 20% of the vegetation. |
| Herbaceous | More than 80% of the vegetation is herbaceous. |
| Pasture/Hay | More than 20% of the vegetation is pasture or hay vegetation for livestock. |
| Cultivated Crops | More than 20% of the vegetation is used for annual crops. |
| Woody Wetlands | More than 20% of the vegetation is forest or shrubland and soil is periodically covered with water. |
| Emergent Herbaceous Wetlands | More than 80% of the vegetation is perennial herbaceous and soil is periodically covered with water. |
