# Supplementary material for: Eco-epidemiology of Rickettsia amblyommatis and Rickettsia parkeri in naturally infected ticks (Acari: Ixodida) from South Carolina
Source: Parasit Vectors. 2024 Jan 25;17:33. doi: 10.1186/s13071-023-06099-z (PMC10811935; doi:10.1186/s13071-023-06099-z)

The following figures depict the plots of the beta estimates variance among the iterations for the variables included in the *R. amblyommatis* and *R. parkeri* analysis.


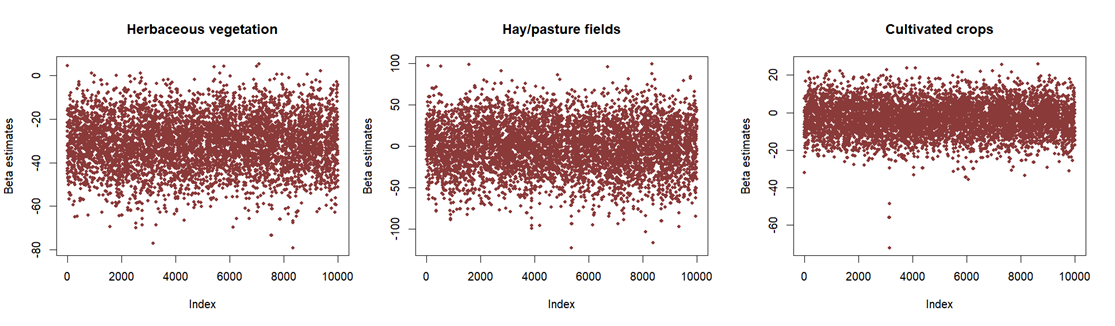
**
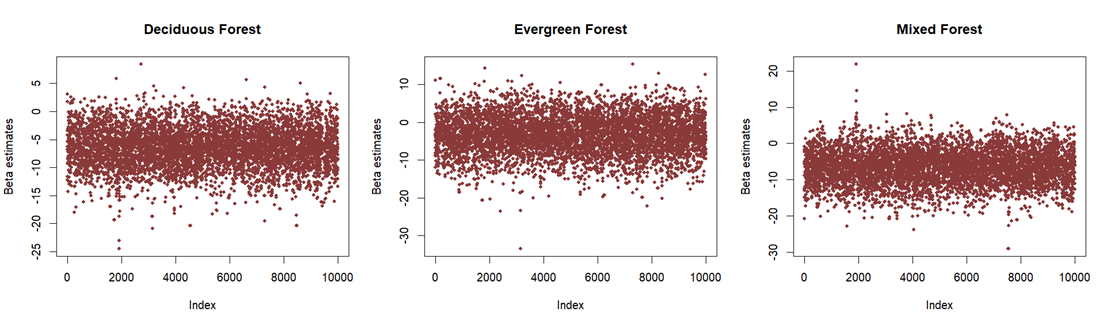

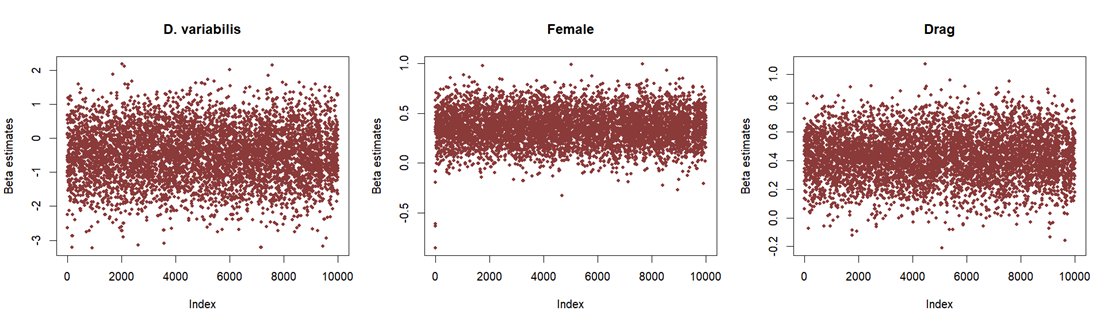
*R. amblyommatis***


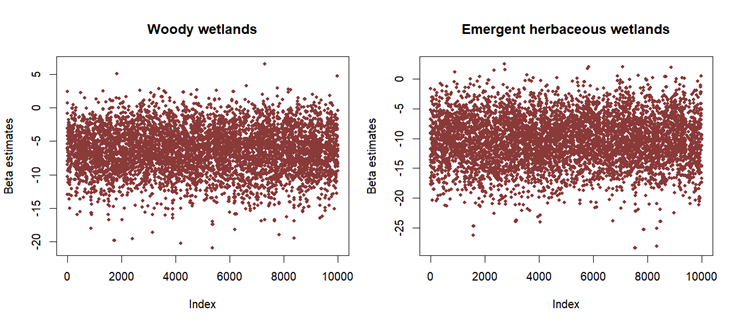


***R. parkeri***

**
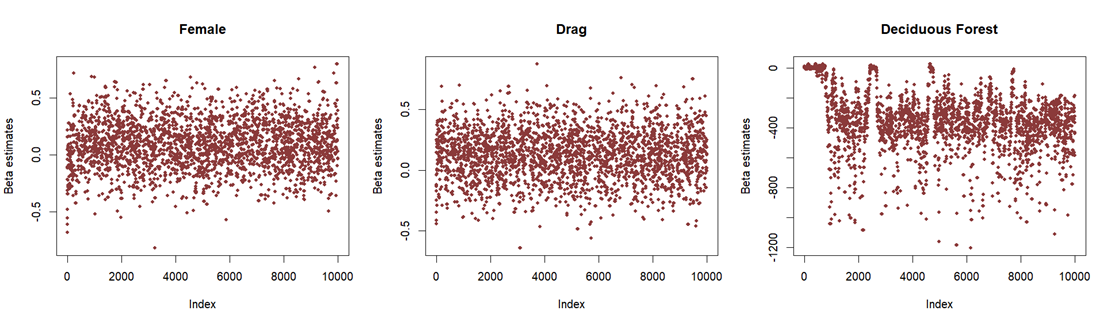
**
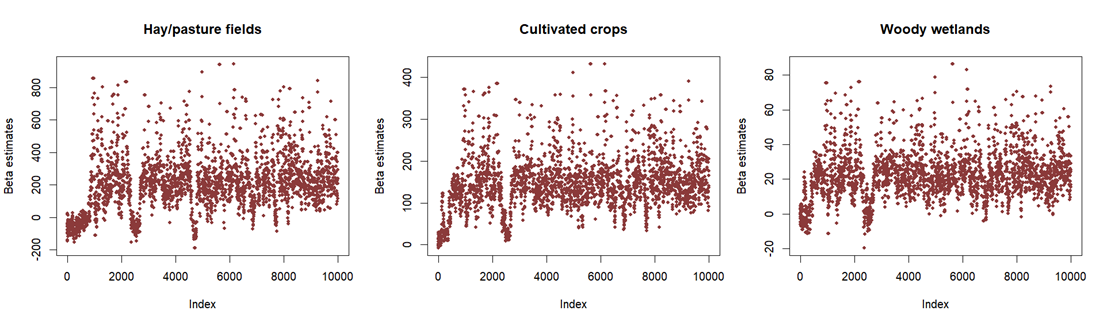


**
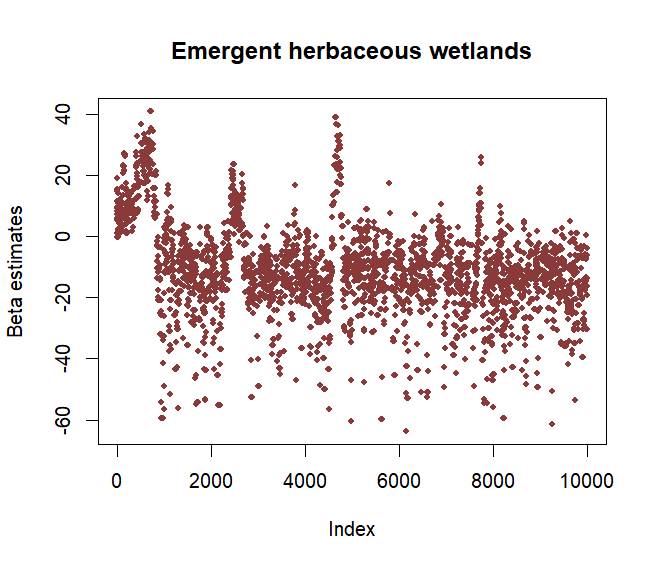
**
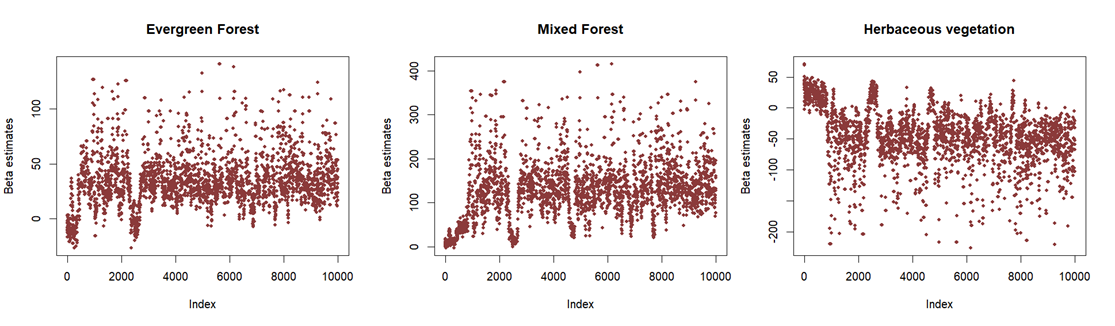

Supplement: Supplementary file 2 — Additional file 2: Markov chain Monte Carlo analysis technical results. [file 13071_2023_6099_MOESM2_ESM.docx]
